# Transcription factor Yin-Yang 2 alters neuronal outgrowth in vitro

**DOI:** 10.1007/s00441-015-2268-7

**Published:** 2015-09-09

**Authors:** Martin Klar, Pascal Fenske, Fanny Rezza Vega, Christof Dame, Anja U. Bräuer

**Affiliations:** Department of Neonatology, Charité – Universitätsmedizin Berlin, Augustenburger Platz 1, 13353 Berlin, Germany; Institute of Cell Biology and Neurobiology, Center for Anatomy, Charité – Universitätsmedizin Berlin, Charitéplatz 1, 10117 Berlin, Germany; Institute of Anatomy, Universitätsmedizin Rostock, Gertrudenstraße 9, 18055 Rostock, Germany

**Keywords:** Differentiation, Neuron, Cell death, Brain, Morphology, Mouse

## Abstract

The Yin-Yang 2 (YY2) protein is the most recently described member of the family of YY transcription factors. Despite its high structural and functional homology with the well-characterized YY1, less is known about its role in biological processes. In previous studies, we have found differential *yy2* mRNA expression levels in various cell types of the murine brain. To investigate the functional implication of yy2 in neurons, we have examined the influence of altered cellular yy2 concentrations during neuronal differentiation. Our results indicate that both the up- and down-regulation of yy2 significantly impairs the outgrowth of the major neurite of primary hippocampal neurons and the numbers of neuronal processes in proximate extensions. Moreover, enhanced expression of wild-type yy2 results in increased cell death, whereas elevated expression levels of a yy2 DNA-binding mutant have no effect on cell viability. Therefore, stringent regulation of the cellular yy2 content might be needed to ensure proper neurite outgrowth and cell vitality.

**Electronic supplementary material:**

The online version of this article (doi:10.1007/s00441-015-2268-7) contains supplementary material, which is available to authorized users.

## Introduction

During brain development, exact temporal and spatial regulation of cellular processes are essential for the proper functional maturation of neuronal connectivities. Neurite outgrowth, axonal guidance, and dendritic development, all of which occur during neuronal differentiation, are crucial cellular events for the establishment of the neuronal network (da Silva and Dotti 2002). In this context, primary cultured hippocampal neurons are a well-characterized system for studying the molecular mechanisms that regulate changes of neurite morphology (Dent et al. 2004; Schwamborn et al. 2006). In vitro, specific stages of neuronal differentiation are characterized by the following morphological characteristics: after attaching to the substrate neurons form lamellipodia (stage 1), and within a few hours several short undifferentiated processes of similar length, called neurites, are formed (stage 2). After 24–48 h, one of these neurites is selected to become the axon and begins to elongate rapidly, whereas the length of the other neurites is not substantially changed (stage 3). Several days later, the remaining neurites begin to grow and differentiate into dendrites (stage 4). The axon and dendrites then reach maturation, and the neurons form synaptic contacts (stage 5; Dotti et al. 1988). The transformation from stage 2 to stage 3 is the crucial step in neuronal differentiation when one neurite becomes the axon, and thereby, the neuron acquires a polar morphology. This process involves the coordinated assembly and activation of a number of signaling molecules and their transcriptional regulatory downstream targets to control axon specification.

The biological impact of the transcription factor Yin-Yang 1 (YY1) in neurogenesis has been intensively studied (He and Casaccia-Bonnefil 2008). However, a distinct functional implication of its homolog YY2 in neuronal development has not yet been described. YY2 shares a variety of structural and functional similarities with YY1. Both proteins recognize an identical DNA-binding element with a highly conserved C-terminal C2H2-zinc finger domain (Kim et al. 2007; Luo et al. 2006; Nguyen et al. 2004). Once bound to DNA, these transcription factors can act as activators or repressors (Usheva and Shenk 1996). Depending on the particular target gene, both YY-proteins can mediate either identical or contrary transcriptional activities (Chen et al. 2010; Klar and Bode 2005).

Without specific antibodies against mouse yy2, the cellular content of this protein was initially estimated by analyzing the content of *yy2* mRNA. At first, the differential expression pattern of *yy2* mRNA in the brain was visualized by in situ hybridization techniques (Luo et al. 2006). For a better understanding of the region- or cell-type-specific *yy2* expression, we performed quantitative polymerase chain reaction (qPCR) expression analyses in the developing murine brain and in neurons, astrocytes, and microglia (Drews et al. 2009). Thereby, we identified spatial and temporal differences in *yy2* expression levels in the neocortex and cerebellum, supporting the specific regulatory functions of yy2 within these tissues at particular developmental stages. Moreover, we found lower *yy2* levels in neurons, compared with astrocytes or microglia.

In our present study, we show that the length of the major neurite is directly deregulated by knockdown or overexpression of the cellular yy2 expression. Furthermore, yy2 overexpression influences the number of neuronal processes in proximate extensions. We have evidence that cofactors are essentially involved in this process. Our experiments imply a functional role of yy2 for proper neurite development.

## Materials and methods

### Animals

Pregnant, postnatal, and adult C57BL/6 mice, obtained from our central animal facility, were kept under standard laboratory conditions (12 h light/dark cycle; 55 ± 15 % humidity; 24 ± 2 °C room temperature [RT] and water ad libidum) in accordance with German and European guidelines (2010/63/EU) for the use of laboratory animals. Approval of experiments was obtained from the local ethical committee of Berlin (LAGeSO: T0108/11).

### Plasmid construction

The mouse *yy2* coding sequence (NCBI accession no. EF688658) was cloned via the *XhoI* and *BamHI* restriction sites into the pCLEG expression vector (Chen et al. 2005). The mouse yy2-FLAG expression plasmid was generated by insertion of a FLAG-linker (5′-cat gga cta caa gga cga cga tga caa gag atc tt-3′ and 5′-cta gaa gat ctc ttg tca tcg tcg tcc ttg tag tcc atg ggc c-3′) into pcDNA3.1(−)muyy2 (Klar and Bode 2005) via *ApaI* and *XbaI* restriction sites. The mouse *yy1* coding sequence (NCBI accession no. NM_009537) was cloned via *BamHI* and *EcoRI* restriction sites into the eukaryotic expression vector pcDNA3.1(−). The resulting plasmids pCLEGmyy2, pcDNA3.1(−)FLAGmyy2, and pcDNA3.1(−)myy1 were subsequently verified by sequencing analysis. The yy2 DNA-binding mutant comprising a T312A substitution, derived from human YY1 mutant T348D (Rizkallah and Hurt 2009), was generated by site-directed mutagenesis with pCLEGmyy2 and the following primer pair: 5′-cag ctt gtt cac gct gga gag aaa cc-3′ and 5′-ggt ttc tct cca gcg tga aca agc tg-3′. Correct amplification was confirmed by Sanger sequencing. For transient knockdown analyses of murine yy2, three different short hairpin RNA (shRNA)-expressing plasmids were constructed. The shRNA sequences were cloned into a *BglII*/*SalI* linearized pCGLH vector (Chen et al. 2005): shRNA-yy2-528 (5′-gat ctc gac aac cta cta ttc agt cct gtt caa gag aca gga ctg aat agt agg ttg ttt ttt tgg aac-3′ and 5′-tcg agt tcc aaa aaa aca acc tac tat tca gtc ctg tct ctt gaa cag gac tga ata gta ggt tgt cga-3′), shRNA-yy2-539 (5′-gat ctc gtt cag tcc tga att tgg aag ctt caa gag agc ttc caa att cag gac tga att ttt tgg aac-3′ and 5′-tcg agt tcc aaa aaa ttc agt cct gaa ttt gga agc tct ctt gaa gct tcc aaa ttc agg act gaa cga-3′), shRNA-yy2-634 (5′-gat ctc gag aga atg gtc aag gtg agc ttt caa gag aag ctc acc ttg acc att ctc ttt ttt tgg aac-3′ and 5′-tcg agt tcc aaa aaa aga gaa tgg tca agg tga gct tct ctt gaa agc tca cct tga cca ttc tct cga-3′). The numbers in the plasmid nomenclature reflect the corresponding position of the first shRNA base pair within the murine *yy2* mRNA sequence (NCBI accession no. NM_001098723). All insertions were verified by sequencing. A pCGLH shRNA expression plasmid against *luciferase* mRNA (shRNA-luc) was used for control experiments (Franke et al. 2012).

### Hippocampal primary neurons: preparation and transfection

Hippocampal primary neurons were isolated from mouse embryos at day 18 (E18). Hippocampi from several embryos were collected, pooled, and washed twice in ice-cold HBSS (Hank’s buffered salt solution, Life Technologies, Darmstadt, Germany). The tissue was incubated in 4 ml HBSS and 400 μl trypsin for 15 min at 37 °C, resuspended in MEM plating medium supplemented with 10 % horse serum, 0.6 % glucose, 100 U/ml penicillin, and 100 μg/ml streptomycin (all Life Technologies). After dissociation, neurons were plated for immunocytochemical experiments at a density of 100,000 cells/cm^2^ onto poly-L-lysine (Sigma-Aldrich, St. Louis, Mo., USA)-coated cover slips in 12-well plates. After 3 h, cells were washed twice with phosphate-buffered saline (1 × PBS) and incubated in Neurobasal A medium, supplemented with 2 % B27, 0.5 mM glutamine, 100 U/ml penicillin, 100 μg/ml streptomycin (all Life Technologies) at 37 °C and 5 % CO_2_. Primary cells were routinely maintained at 37 °C with 5 % CO_2_ in Neurobasal A medium supplemented as described above. Primary neurons were transfected at division 1 (DIV1) by using Effectene (Qiagen, Hilde, Germany) and analyzed 48 h later (DIV3).

### Immunocytochemistry and microscopy

Primary neurons were fixed 48 h after transfection in ice-cold 4 % paraformaldehyde with 15 % sucrose in PBS for 10 min at RT, washed three times with 1 × PBS for 10 min, permeabilized with 0.1 % Triton X-100, 0.1 % sodium citrate in PBS for 3 min at 4 °C, washed again three times with 1 × PBS for 10 min, and incubated for 1 h with blocking solution, at RT, containing 10 % FCS in 1 × PBS. Neurons were incubated with primary antibodies overnight at 4 °C and then washed three times with 1 × PBS for 10 min. Antibodies were used at the following dilutions: anti-green fluorescent protein (GFP; AbCam, Cambridge, UK) 1:2500, anti-FLAG (7425; Sigma-Aldrich) 1:1000, and anti-YY2 (A-5; sc-377008; mouse monoclonal antibody; Santa Cruz Biotechnology, Heidelberg, Germany) 1:100 and 1:250, respectively. The secondary antibodies, namely Alexa Fluor 488 and Alexa Fluor 568 at a dilution of 1:1500, were subsequently applied for 90 min at RT. Slides were mounted with Immu-Mount (Thermo Fisher Scientific, Waltham, Mass., USA). Confocal images were acquired with an upright laser microscope (Leica DM 2500) equipped with a ×63 objective (oil immersion 1.4 NA) and a ×40 objective (oil immersion, 1.25 NA) by using the 488-nm line of an argon-ion laser and the 543-nm line of a helium-neon laser. Background correction and adjustment of brightness and contrast were performed by using Leica confocal software. For co-localization studies, z-stacks of both fluorophores were sequentially acquired and projections averaged.

### Morphological analysis

For morphological analysis, fluorescence images of primary neurons were acquired with an inverted epifluorescence microscope (Olympus IX 81) equipped with a ×100 objective (oil-immersion, 1.3 NA). Images were taken by camera F-View II (Olympus) by using cellSens DimensionDesktop Version 1.4.1 (Build 8624) software from Olympus. Background correction and the adjustment of brightness and contrast were performed by using Adobe® Photoshop CS 3 software. The length of the longest neurite was measured by using ImageJ 1.45 s software. Data analysis was performed by using GraphPad Prism 5 (GraphPad Software, La Jolla, Calif., USA) on *n* = 48 for the control (pCLEG empty vector) and yy2 wild-type (wt), *n* = 54 for yy2 mut, *n* = 29 for shRNA-luc, and *n* = 33 for shRNA-yy2-539. Numbers of primary neurites emerging from the cell body were calculated. Data analysis was performed by using GraphPad Prism 5 from *n* = 46 for control (pCLEG empty vector) and yy2 wt, *n* = 52 for yy2 mut, *n* = 27 for shRNA-luc, and *n* = 31 for shRNA-yy2-539. The number of neurites was determined by means of Sholl analysis, by using 24 10-μm-spaced concentric circles with maximal distances of 250 μm distal to the cell body. The innermost circle with a radius of 20 μm was placed onto each neuron, with its center roughly coinciding with the center of the soma. Neurite intersections at each circle were manually counted by using ImageJ. Sholl analysis of neurons was performed by using GraphPad Prism 5 on *n* = 48 for control (pGLEG empty vector) and yy2 wt, *n* = 54 for yy2 mut, *n* = 29 for shRNA-luc, and *n* = 33 for shRNA-yy2-539. One investigator, blind to the experimental conditions, performed the measurement. For all measurements, 3–6 different transfections were used. The measurement performed in micrometers or n was converted to a percentage. Data are reported and presented as means ± SEM. Statistical significance was determined by using the two-tailed Mann Whitney test for unpaired data at the given significance level (*P*: *** 0.001 < *P*; ** 0.001 < *P* < 0.01; * 0.01 < *P* < 0.05).

### Cell lines and transfection experiments

N1E-115 mouse neuroblastoma (ATCC CRL-2263), human embryonic kidney HEK293 (DSMZ no.: ACC 305), and human cervix carcinoma HELA (DSMZ no.: ACC 57) cells were grown at 37 °C in a humidified 5 % CO_2_-enriched atmosphere in Dulbecco’s modified Eagle’s medium (DMEM) supplemented with 10 % fetal bovine serum, 50 U/ml penicillin, and 10 mg/ml streptomycin (all PAN-Biotech, Aidenbach, Germany). For transfection experiments, cells were plated at a density of 50,000 cells/ml per cm^2^ onto poly-L-lysine (Sigma-Aldrich)-coated coverslips in 12-well plates. Next day, the medium was renewed, and the cells were transfected with 1 μg pCLEG, pCLEG-yy2, or pCLEGmYY2 (T312A) by using lipofection reagent FuGene 6 (Roche, Mannheim, Germany) according to manufacturer’s protocol and analyzed 24 h later. Transfection experiments to validate shRNA-expression constructs were conducted with 200,000 HEK 293 cells per 6-well plate by lipofection with Metafectene (Biontex, München, Germany). One day after plating, cells were transfected with 24 μg of the particular pCGLH construct. Next day, cells were transfected with 1 μg of the corresponding pCGLH and 1 μg yy2 expression plasmid pCLEGmyy2, respectively. After 24 h, cells were lyzed in RIPA buffer (0.05 M TRIS, pH 7.4, 0.15 M NaCl, 0.001 M EDTA, 0.001 M EGTA, 1 % Triton-X, 0.25 % [Na]DOC). Ectopic expression of mouse yy2 wild-type and T312 mutant protein in HELA cells was achieved by using the Metafectene transfection protocol. At 2 days after transfection with 2 μg pCLEGmyy2 or pCLEGmyy2 (T312A) plasmid, respectively, cells were lyzed in RIPA buffer.

### Western blot analysis

Whole cell lysates were prepared by lysis in RIPA buffer. Mouse yy2 and yy1 were in vitro-translated from corresponding pcDNA3.1(−) expression plasmids by using the TNT-Coupled Transcription/Translation system (Promega, Mannheim, Germany) according the manufacturer’s protocol. Whole cell lysates or in vitro-translated proteins were separated by SDS-polyacrylamide gel electrophoresis and subsequently transferred by semi-dry blot technique onto nitrocellulose membranes. After the membrane had been blocked overnight in 5 % nonfat dry milk/1 × TBS-T (30 mM TRIS-HCl pH 7.6, 150 mM NaCl, 0.1 % Tween 20), immobilized proteins were detected by using the following primary and horseradish-peroxidase-conjugated secondary antibodies: anti-FLAG (Sigma-Aldrich; M2; 1:2000), anti-β-actin (Sigma-Aldrich; 1:5000), anti-GFP (Abcam, 1:5000), anti-YY1 (sc-281; 1:500), anti-YY2 (sc-377008; 1:500), anti-mouse (sc-2005; 1:10000), and anti-rabbit (sc-2004; 1:10,000; all from Santa Cruz Biotechnology). Visualization was conducted with the Western Blotting Luminol Reagent (Santa Cruz Biotechnology).

### Propidium iodide staining

Cell survival was analyzed by using the intercalating agent propidium iodide (PI; Carl Roth, Karlsruhe, Germany). At 24 h after transfection of N1E-115 cells, the culture medium was replaced with fresh preheated medium containing 5 μg/ml PI and incubated for 1 h at 37 °C under a humidified 5 % CO_2_-enriched atmosphere. Fluorescence images were acquired with the inverted Olympus IX 81, as described above, but equipped with a ×20 objective (0.45 NA). Each experiment was carried out in triplicate and repeated 6–9 times. PI-stained cells were counted by using ImageJ Gel densitometry software. The percentage of dead cells was calculated as a proportion of PI-positive cells to that of total cells. The amount of total cells per well were calculated based on the plated cell number. Data analysis was performed by using GraphPad Prism 5 on *n* = 27 for control (pCLEG empty vector), *n* = 24 for yy2 wt, and *n* = 52 for yy2 mut. Data are reported and presented as means ± SEM. Significance was assessed by using the two-tailed Mann Whitney test for unpaired data at the given significance level (*P*: *** 0.001 < *P*; ** 0.001 < *P* < 0.01; * 0.01 < *P* < 0.05.

### Quantitative reverse transcription plus PCR

N1E-115 cells were transiently transfected with empty pCLEG (control), yy2 wt or yy2 mut expression plasmids. After 24 h, total RNA was isolated by using the TRIzol protocol (Life Technologies). Subsequently, 2 μg RNA were reversely transcribed into cDNA and subjected to qPCR analysis by using the following TaqMan Gene Expression Assays for *β-actin* (4352933E), *yy2* (Mm03059489_sH), *bax* (Mm00432051_m1), and *bcl2* (Mm00477631_m1; all Life Technologies).

## Results and discussion

### YY2 is expressed in immature neurons

**Fig. 1:**
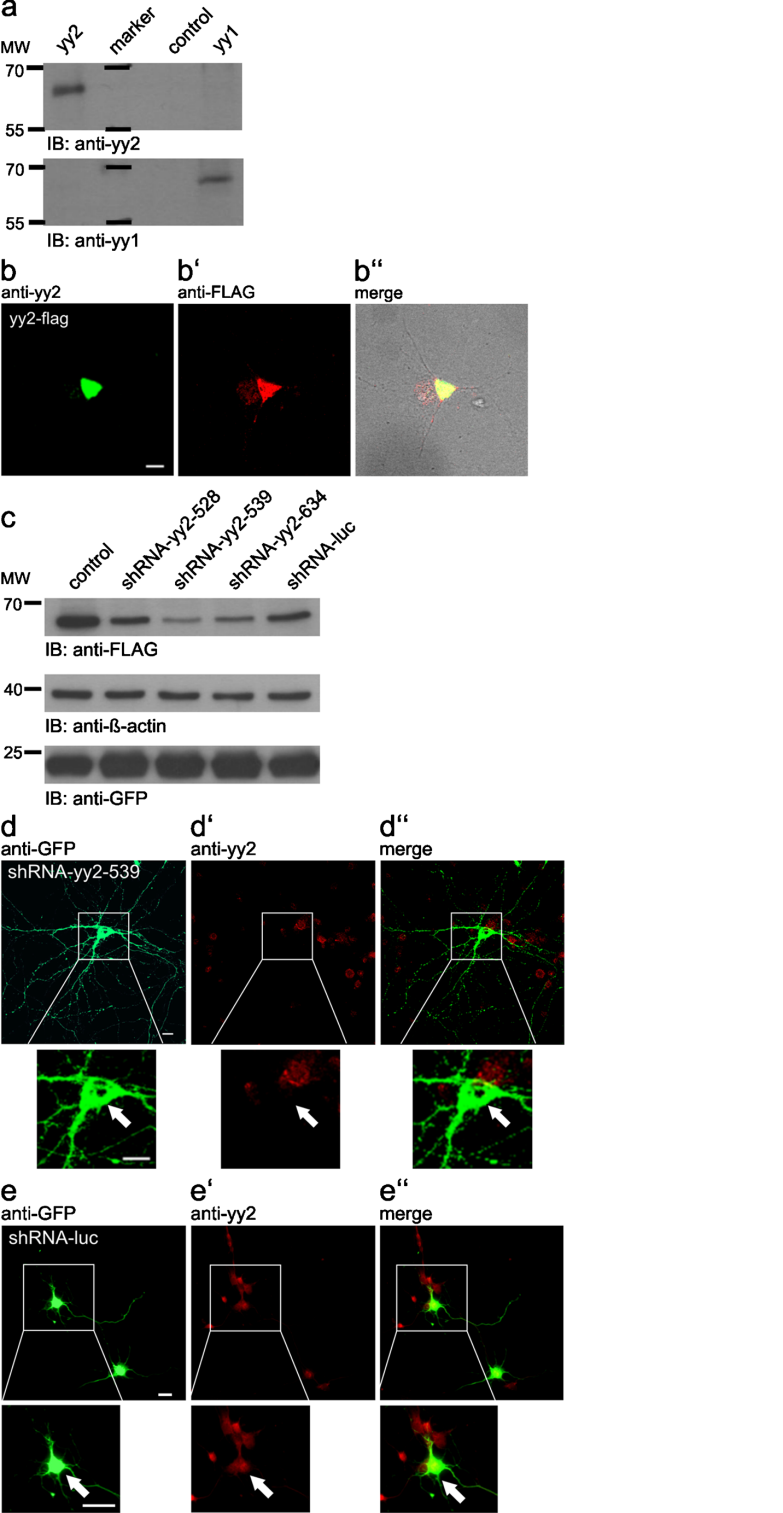
Antibody and yy2 knockdown verification. **a** Immunoblot (*IB*) analysis for testing reactivity and specificity of the anti-yy2 antibody sc-377008 by using in vitro-translated mouse yy1 and yy2, respectively. Pure reticulocyte lysate served as control. **b**, **b’**, **b’’** Primary neurons were transfected with FLAG-tagged yy2, and confocal fluorescence microscopy was conducted with indicated antibodies. **c** Knockdown efficiency test of three different short hairpin RNA (shRNA) expression plasmids directed against mouse *yy2* in HEK293 cells. Co-expression of FLAG-tagged yy2 was used to identify knockdown efficiencies easily. An shRNA expression plasmid against *luciferase* mRNA (*shRNA-luc*) and the empty vector construct (*control*) were used for control experiments. A Western blot against green fluorescent protein (*GFP*) was performed to compare transfection efficiencies of the shRNA expression plasmids and the immunological detection of β-actin, which served as a loading control. **d**, **d’**, **d’’** Visualization of endogenous yy2 and its efficient knockdown in primary neurons compared with non-transfected and control transfected (**e**, **e’**, **e’’**) cells via confocal fluorescence microscopy. Co-localization analysis of GFP/shRNA expression plasmid and yy2 was conducted by using the indicated antibodies. The areas in the *white boxes* are shown at higher magnification *below* the relevant micrograph (*arrow* cell of interest). *Bars* 50 μm

Here, we investigated the functional implication of yy2 during neuronal differentiation by deregulation of the endogenous yy2 protein content. To date, the yy2 transcription factor has not been studied in mice at the protein level at all. Thus, we initially validated the commercially available anti-YY2 mouse monoclonal antibody from Santa Cruz Biotechnology for immunoblots and for immunofluorescence microscopy analysis to visualize altered cellular yy2 concentrations. Because of the high homology of both yy proteins, especially within the C-terminal DNA-binding zinc finger domain, some purchasable antibodies cross-react between both yy transcription factors (Klar 2010, 2012; Klar and Bode 2005). To establish the specificity of the monoclonal antibody, we performed immunoblots with in vitro-translated yy2 and yy1 proteins. As depicted in Fig. 1a, the anti-yy2 antibody exclusively detected yy2, whereas yy1 was only recognized by a specific anti-yy1 antibody. To investigate further the suitability of this anti-yy2 antibody for immunofluorescence, we transfected hippocampal cultured neurons with expression plasmids encoding for FLAG-tagged yy2 and performed co-localization studies (Fig. 1b, anti-yy2, b’ anti-FLAG, b’’ merge). This analysis clearly demonstrated that the yy2 antibody recognized FLAG-yy2 overexpression in primary neurons.

To examine the effects of an altered cellular content of yy2 in immature neurons, we generated molecular tools for ectopic expression and knockdown studies of yy2 optimized for detection by fluorescence microscopy. Next to yy2 itself or a particular shRNA, all constructs constitutively co-expressed enhanced GFP (eGFP) for the identification of transfected primary neurons. Unfortunately, the reactivity of commercially available antibodies against mouse yy2 was not sufficient to detect the endogenous level of this transcription factor in whole-cell and nuclear extracts from hippocampal primary neurons by Western blot analysis. To validate the knockdown efficiencies of three different shRNA expression constructs, HEK293 cells were co-transfected with FLAG-tagged mouse yy2. As shown in Fig. 1c, construct shRNA-539 mediated a significantly reduced expression of FLAG-yy2. Finally, in order to confirm the knockdown capacity of shRNA-539 in primary neurons, we next targeted cellular yy2 in primary hippocampal neurons. As depicted in Fig. 1d, d’, d’’, we clearly achieved an efficient knockdown of endogenous yy2, whereas all non-transfected neurons or control transfected neurons showed clear nuclear yy2 staining (Fig. 1e, e’, e’’).

Taken together, our data, which were obtained by using a commercially available anti-yy2 antibody, were able first to demonstrate the specificity of this antibody and second to visualize endogenous yy2 and its shRNA-driven transient knockdown in primary hippocampal neurons.

### Changes of endogenous yy2 expression influence primary neuron differentiation

**Fig. 2:**
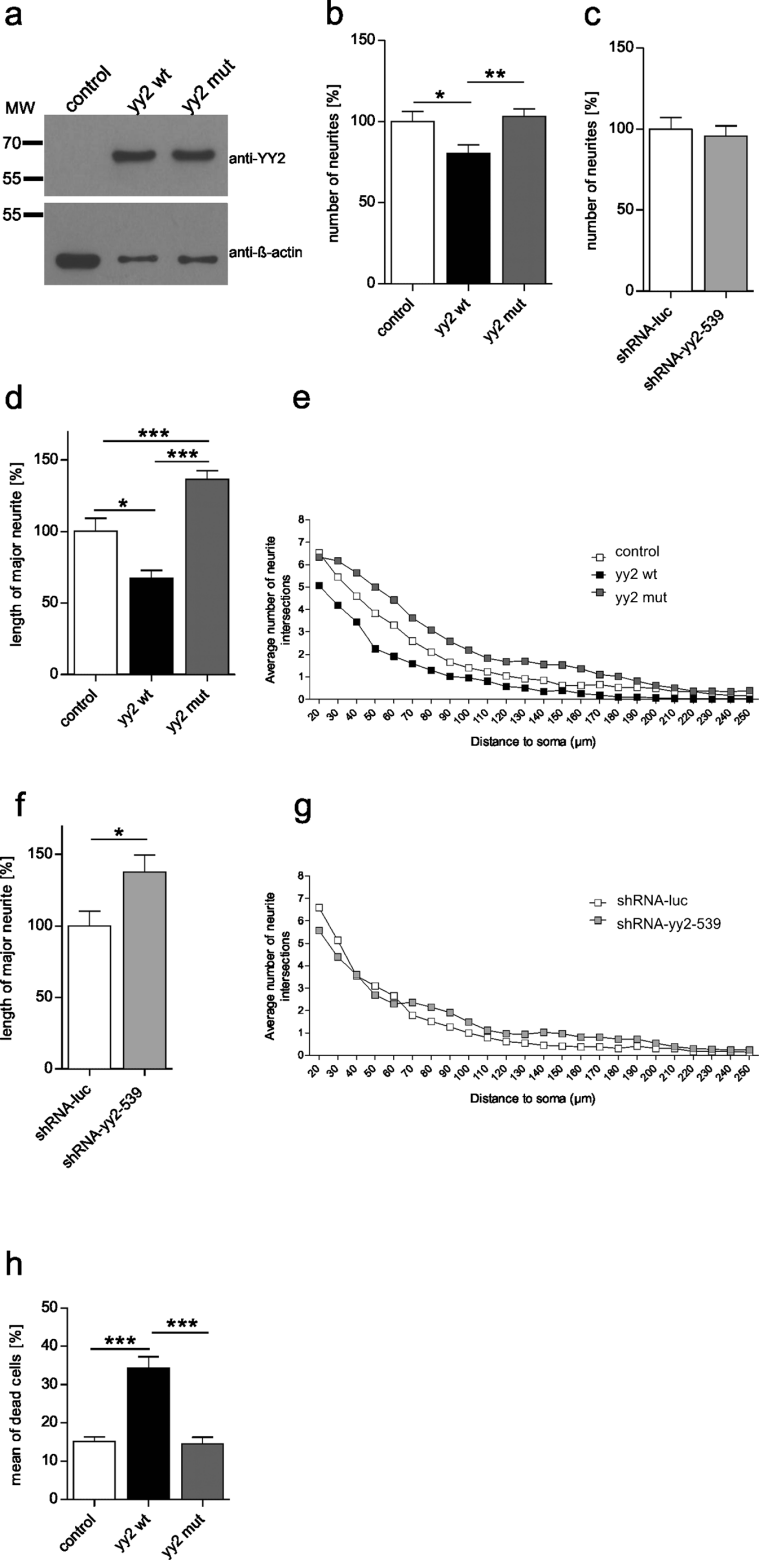
Morphological analyses of primary neurons with altered yy2 expression levels. **a** Expression control experiments in HELA cells revealed proper ectopic protein expression of wild-type (*yy2 wt*) and mutant (*yy2 mut*) yy2, respectively. Western blot against β-actin served as loading control. **b**, **c** Comparison of the number of neurites per neuron with respect to yy2 wt or yy2 mut overexpression and yy2 knockdown in primary hippocampal neurons. Investigations on the impact of yy2 overexpression levels on the outgrowth of major neurites (**d**) and the numbers of neuronal processes (**e**) in primary hippocampal neurons and yy2 knockdown effects on the outgrowth of the major neurites (**f**) and the numbers of neuronal processes (**g**). **h** PI-staining. Quantification of dead N1E-115 neuroblastoma cells transiently transfected with yy2 wt and yy2 mut, respectively

In early, not yet polarized, hippocampal cultured neurons, we investigated whether the expression of yy2 influenced neurite outgrowth. Initial expression control experiments in HELA cells of both wild-type yy2 (yy2 wt) and a mutant (yy2 mut), which is unable to bind to DNA, revealed proper ectopic protein production (Fig. 2a). In transiently transfected primary hippocampal neurons, yy2 overexpression resulted in a decrease in the number of neurites projecting to the soma by 20 % (Fig. 2b), whereas overexpression of the mutant yy2 did not show such changes (Fig. 2b). In addition, the number of neurites projecting to the soma was not significantly altered by the knockdown of yy2 (Fig. 2c). All these findings implied that a normal or reduced endogenous yy2 concentration did not impact the number of neurites formed by a single neuron, whereas an elevated yy2 level seemed to compete with another transcription factor, possibly YY1, for genomic DNA-binding sites relevant for the regulation of a proper number of neurites. Of note, the latter observation is reminiscent of the effects previously published for yy2 in reporter gene analyses. In these experiments, some gene promoters were activated in response to lower concentrations of overexpressed yy2, whereas higher yy2 expression level resulted in decreased activities of the same promoters (Nguyen et al. 2004). The transcriptional activities of yy2 have been proposed to be promoter- and dose-dependent and putatively dependent on other transcription factors such as yy1.

Moreover, a higher expression level of the yy2 transcription factor leads to a decrease in the length of the longest neurite, which later will become an axon (Dotti et al. 1988). Here, we have demonstrated that the longest neurite reaches only 67 % of the length of the control cells (Fig. 2d, Supplemental Fig. 1a, a’). Interestingly, the overexpression of the yy2 DNA-binding mutant shows an increase of 36 % in the length of the longest neurite compared with the control group (Fig. 2d, Supplemental Fig. 1a’’). Moreover, Sholl analyses also revealed substantially reduced numbers of neuronal processes in proximate extensions in yy2 wt overexpressing primary neurons, whereas the yy2 DNA-binding mutant shows the opposite effect (Fig. 2e, Supplemental Table 1, supplemental Table 3). However, the knockdown of yy2 also leads to a 37 % increase of the length of the longest neurite compared with that of the control cells (Fig. 2f, supplemental Fig. 1b, b’) but does not show significant changes in neurite branching (Fig. 2g, Supplemental Table 2). In contrast to the examinations mentioned above regarding the influence of the altered yy2 level on the absolute number of neurites, the length of the major neurite is directly deregulated by changes of the cellular yy2 content. In particular the enhanced length growth of neurites observed in the knockdown experiments underlines the functional role for yy2 during neuron differentiation, whereas a similar effect achieved by the overexpression of the yy2 DNA-binding mutant suggests that cofactors are essentially involved in the yy2-mediated control of neurite length growth. The excess of mutant yy2 seems to subdue the interaction between DNA-bound yy2 and its interacting protein partners. So far, only a few yy2-interacting proteins have been described, namely *ring finger protein 1* and *2* (RING1, RING2) and *embryonic ectoderm development* (EED), all of which belong to the family of Polycomb-group (PcG) proteins (Garcia-Tunon et al. 2011). As PcG proteins are known to play a fundamental role mainly in gene silencing, we are tempted to speculate that yy2 is part of this gene regulatory machinery. Interestingly, knockdown studies of *enhancer of zeste 2* (EZH2), which represents next to EED and *suppressor of zeste 12 homolog* (SUZ12), one of the three core subunits of the polycomb repressor complex 2 (PRC2; Cao et al. 2002) by Wang et al. (2012) have shown that a decreased EZH2 level induces neurite extensions. All these results indicate that changes in the expression level of yy2 negatively influence neuronal differentiation processes.

### YY2 overexpression leads to neuronal cell death

During our transient yy2 transfection assays in primary hippocampal neurons, we found many obviously degraded cells when wild-type yy2 was overexpressed (data not shown). Thus, we asked whether an increase of yy2 expression specifically forces neuronal cell death. By using the neuroblastoma cell line N1E-115 in order to attain a higher transfection rate compared with primary neurons, we found that yy2 wt overexpression significantly increased cell death compared with that of controls (control = 14 %; yy2 wt = 34 %; Fig. 2h). Interestingly, elevated level of yy2 mut had no influence on cell death (Fig. 2h). Functionally to associate the increased cell death with apoptosis, we subsequently analyzed, via qPCR, whether the common (anti-) apoptotic genes *Bax* and *Bcl2* were deregulated. Although yy2 wt and yy2 mut were strongly upregulated, both *Bax* and *Bcl2* showed no alterations at all. Thus, the observed wild-type yy2-induced-cell death is not caused by classical apoptosis (Supplemental Fig. 1c). All these findings indicate that an increased yy2 level forces cell death in neuronal cells as a consequence of direct yy2-DNA interaction. We have to assume that an excess of yy2 competes with other transcription factors for functional DNA-binding sites of pivotal genes.

In summary, our experiments support a functional role for yy2 for proper neurite development. A strict regulation of the cellular yy2 concentrations seems to be needed to ensure normal neurite outgrowth and cell vitality.

## Electronic supplementary material

Supplemental Fig. 1 Fluorescence images of representative transfected primary neurons showing morphological alterations in length and number of neurites. **a–a’’** Overexpression of empty GFP vector as a control (**a**), wild type yy2 (**a’**), and mutant yy2 (**a’’**). **b** Transfected neurons with shRNA-luc as control and yy2 knockdown with shRNA-yy2-539 (**b’**). **c** Expression analyses in murine N1E-115 neuroblastoma cells of *bax* and *bcl2* transcripts in response to transient overexpression of wild-type or mutant yy2. All indicated mRNA data were normalized to *β-actin* transcript levels. *Bar* 50 μm. (GIF 83 kb)

High resolution image file (TIFF 12924 kb)

Supplemental Table 1 SPSS statistics of the Sholl analysis from Fig. 2e (DOC 97 kb)

Supplemental Table 2 SPSS statistics of the Sholl analysis from Fig. 2g (DOC 124 kb)

Supplemental Table 3 Data from the multiple comparisons Bonferroni statistics of Fig. 2e. (DOC 248 kb)
