# Supplementary figures and images for: Transcription factor Yin-Yang 2 alters neuronal outgrowth in vitro
Source: Cell Tissue Res. 2015 Sep 9;362(2):453–60. doi: 10.1007/s00441-015-2268-7 (PMC4657790; doi:10.1007/s00441-015-2268-7)

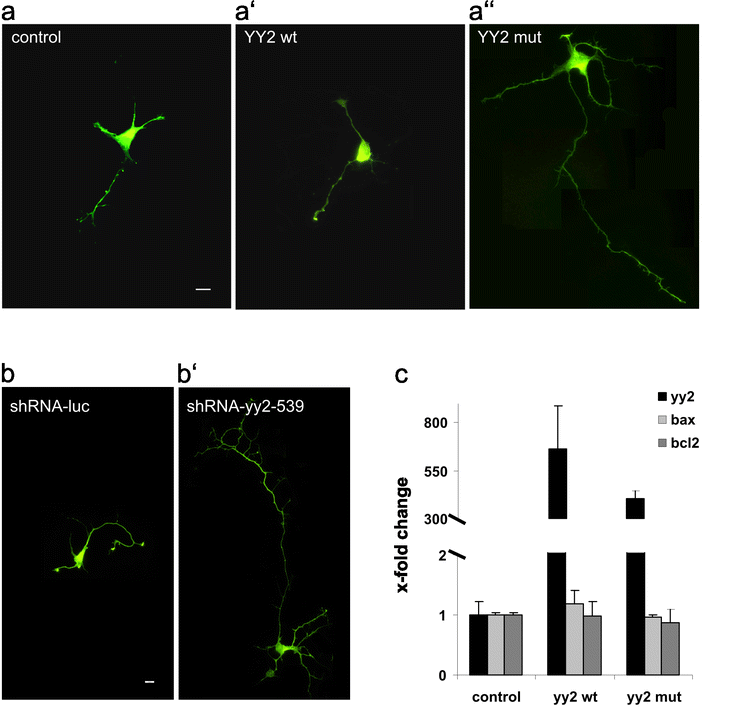

Supplement: Supplementary file 1 — Fluorescence images of representative transfected primary neurons showing morphological alterations in length and number of neurites. a–a’’ Overexpression of empty GFP vector as a control (a), wild type yy2 (a’), and mutant yy2 (a’’). b Transfected neurons with shRNA-luc as control and yy2 knockdown with shRNA-yy2-539 (b’). c Expression analyses in murine N1E-115 neuroblastoma cells of bax and bcl2 transcripts in response to transient overexpression of wild-type or mutant yy2. All indicated mRNA data were normalized to β-actin transcript levels. Bar 50 μm. (GIF 83 kb) [file 441_2015_2268_Fig3_ESM.gif]

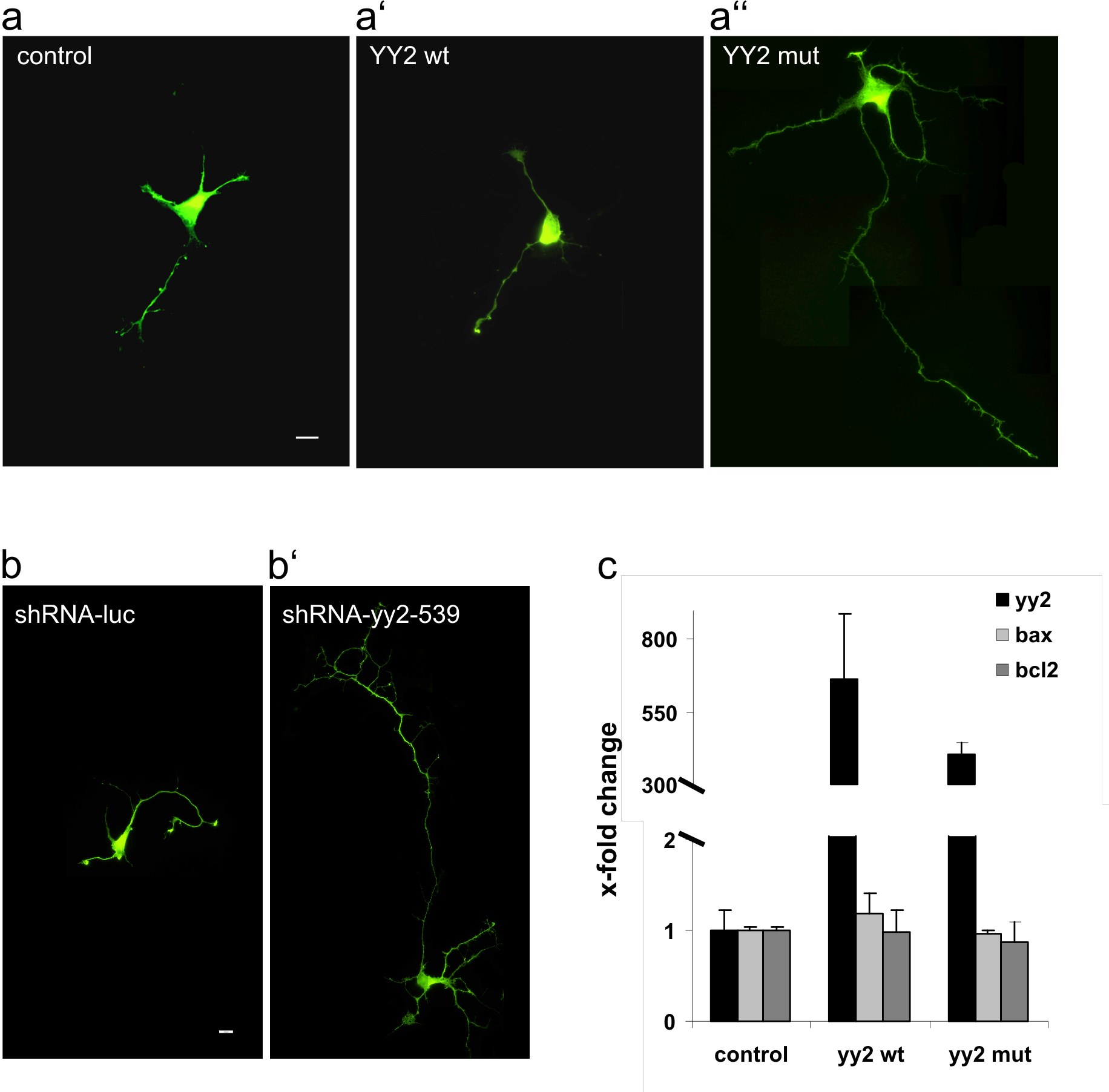

Supplement: Supplementary file 2 — High resolution image file (TIFF 12924 kb) [file 441_2015_2268_MOESM1_ESM.tif]
