# Supplementary material for: Transcription factor Yin-Yang 2 alters neuronal outgrowth in vitro
Source: Cell Tissue Res. 2015 Sep 9;362(2):453–60. doi: 10.1007/s00441-015-2268-7 (PMC4657790; doi:10.1007/s00441-015-2268-7)
Supplement: Supplementary file 3 — SPSS statistics of the Sholl analysis from Fig. 2e (DOC 97 kb) [file 441_2015_2268_MOESM2_ESM.doc]

**ANOVA – overexpression analysis (SPSS Statistics)**

df = degrees of freedom

F = F ratio

Sig. = significance of the F ratio (= p value)

|  | | **Sum of Squares** | **df** | **Mean Square** | **F** | **Sig.** |
| --- | --- | --- | --- | --- | --- | --- |
| Distance 20 | Between Groups | 70,428 | 2 | 35,214 | 4,963 | ,008 |
|  | Within Groups | 1043,072 | 147 | 7,096 |  |  |
|  | Total | 1113,500 | 149 |  |  |  |
| Distance 30 | Between Groups | 115,346 | 2 | 57,673 | 10,207 | ,000 |
|  | Within Groups | 830,627 | 147 | 5,651 |  |  |
|  | Total | 945,973 | 149 |  |  |  |
| Distance 40 | Between Groups | 131,048 | 2 | 65,524 | 10,809 | ,000 |
|  | Within Groups | 891,146 | 147 | 6,062 |  |  |
|  | Total | 1022,193 | 149 |  |  |  |
| Distance 50 | Between Groups | 219,435 | 2 | 109,718 | 24,218 | ,000 |
|  | Within Groups | 665,958 | 147 | 4,530 |  |  |
|  | Total | 885,393 | 149 |  |  |  |
| Distance 60 | Between Groups | 162,417 | 2 | 81,208 | 15,566 | ,000 |
|  | Within Groups | 766,917 | 147 | 5,217 |  |  |
|  | Total | 929,333 | 149 |  |  |  |
| Distance 70 | Between Groups | 106,264 | 2 | 53,132 | 13,929 | ,000 |
|  | Within Groups | 560,729 | 147 | 3,814 |  |  |
|  | Total | 666,993 | 149 |  |  |  |
| Distance 80 | Between Groups | 86,481 | 2 | 43,240 | 15,018 | ,000 |
|  | Within Groups | 423,259 | 147 | 2,879 |  |  |
|  | Total | 509,740 | 149 |  |  |  |
| Distance 90 | Between Groups | 65,268 | 2 | 32,634 | 17,248 | ,000 |
|  | Within Groups | 278,125 | 147 | 1,892 |  |  |
|  | Total | 343,393 | 149 |  |  |  |
| Distance 100 | Between Groups | 42,043 | 2 | 21,022 | 10,923 | ,000 |
|  | Within Groups | 282,917 | 147 | 1,925 |  |  |
|  | Total | 324,960 | 149 |  |  |  |
| Distance 110 | Between Groups | 43,865 | 2 | 21,932 | 8,898 | ,000 |
|  | Within Groups | 362,329 | 147 | 2,465 |  |  |
|  | Total | 406,193 | 149 |  |  |  |
|  | | **Sum of Squares** | **df** | **Mean Square** | **F** | **Sig.** |
| Distance 120 | Between Groups | 45,240 | 2 | 22,620 | 9,840 | ,000 |
|  | Within Groups | 337,933 | 147 | 2,299 |  |  |
|  | Total | 383,173 | 149 |  |  |  |
| Distance 130 | Between Groups | 44,109 | 2 | 22,054 | 11,507 | ,000 |
|  | Within Groups | 281,731 | 147 | 1,917 |  |  |
|  | Total | 325,840 | 149 |  |  |  |
| Distance 140 | Between Groups | 45,015 | 2 | 22,508 | 12,777 | ,000 |
|  | Within Groups | 258,958 | 147 | 1,762 |  |  |
|  | Total | 303,973 | 149 |  |  |  |
| Distance 150 | Between Groups | 37,521 | 2 | 18,760 | 11,991 | ,000 |
|  | Within Groups | 229,979 | 147 | 1,564 |  |  |
|  | Total | 267,500 | 149 |  |  |  |
| Distance 160 | Between Groups | 33,302 | 2 | 16,651 | 14,290 | ,000 |
|  | Within Groups | 171,292 | 147 | 1,165 |  |  |
|  | Total | 204,593 | 149 |  |  |  |
| Distance 170 | Between Groups | 21,572 | 2 | 10,786 | 10,409 | ,000 |
|  | Within Groups | 152,322 | 147 | 1,036 |  |  |
|  | Total | 173,893 | 149 |  |  |  |
| Distance 180 | Between Groups | 21,157 | 2 | 10,579 | 12,869 | ,000 |
|  | Within Groups | 120,843 | 147 | ,822 |  |  |
|  | Total | 142,000 | 149 |  |  |  |
| Distance 190 | Between Groups | 13,054 | 2 | 6,527 | 8,848 | ,000 |
|  | Within Groups | 108,440 | 147 | ,738 |  |  |
|  | Total | 121,493 | 149 |  |  |  |
| Distance 200 | Between Groups | 9,902 | 2 | 4,951 | 7,843 | ,001 |
|  | Within Groups | 92,792 | 147 | ,631 |  |  |
|  | Total | 102,693 | 149 |  |  |  |
| Distance 210 | Between Groups | 6,264 | 2 | 3,132 | 5,480 | ,005 |
|  | Within Groups | 84,009 | 147 | ,571 |  |  |
|  | Total | 90,273 | 149 |  |  |  |
| Distance 220 | Between Groups | 4,177 | 2 | 2,089 | 5,828 | ,004 |
|  | Within Groups | 52,683 | 147 | ,358 |  |  |
|  | Total | 56,860 | 149 |  |  |  |
|  | | **Sum of Squares** | **df** | **Mean Square** | **F** | **Sig.** |
| Distance 230 | Between Groups | 3,122 | 2 | 1,561 | 5,458 | ,005 |
|  | Within Groups | 42,051 | 147 | ,286 |  |  |
|  | Total | 45,173 | 149 |  |  |  |
| Distance 240 | Between Groups | 2,494 | 2 | 1,247 | 5,143 | ,007 |
|  | Within Groups | 35,646 | 147 | ,242 |  |  |
|  | Total | 38,140 | 149 |  |  |  |
| Distance 250 | Between Groups | 3,155 | 2 | 1,577 | 5,763 | ,004 |
|  | Within Groups | 40,238 | 147 | ,274 |  |  |
|  | Total | 43,393 | 149 |  |  |  |
