# Supplementary material for: Transcription factor Yin-Yang 2 alters neuronal outgrowth in vitro
Source: Cell Tissue Res. 2015 Sep 9;362(2):453–60. doi: 10.1007/s00441-015-2268-7 (PMC4657790; doi:10.1007/s00441-015-2268-7)
Supplement: Supplementary file 4 — SPSS statistics of the Sholl analysis from Fig. 2g (DOC 124 kb) [file 441_2015_2268_MOESM3_ESM.doc]

**ANOVA – knockdown analysis (SPSS Statistics)**

df = degrees of freedom

F = F ratio

Sig. = significance of the F ratio (= p value)

|  | | **Sum of Squares** | **df** | **Mean Square** | **F** | **Sig.** |
| --- | --- | --- | --- | --- | --- | --- |
| Distance 20 | Between Groups | 15,760 | 1 | 15,760 | 2,909 | ,093 |
|  | Within Groups | 325,095 | 60 | 5,418 |  |  |
|  | Total | 340,855 | 61 |  |  |  |
| Distance 30 | Between Groups | 8,544 | 1 | 8,544 | 2,216 | ,142 |
|  | Within Groups | 231,327 | 60 | 3,855 |  |  |
|  | Total | 239,871 | 61 |  |  |  |
| Distance 40 | Between Groups | ,046 | 1 | ,046 | ,012 | ,914 |
|  | Within Groups | 231,051 | 60 | 3,851 |  |  |
|  | Total | 231,097 | 61 |  |  |  |
| Distance 50 | Between Groups | 2,550 | 1 | 2,550 | ,716 | ,401 |
|  | Within Groups | 213,659 | 60 | 3,561 |  |  |
|  | Total | 216,210 | 61 |  |  |  |
| Distance 60 | Between Groups | 1,914 | 1 | 1,914 | ,647 | ,424 |
|  | Within Groups | 177,521 | 60 | 2,959 |  |  |
|  | Total | 179,435 | 61 |  |  |  |
| Distance 70 | Between Groups | 5,024 | 1 | 5,024 | 1,769 | ,189 |
|  | Within Groups | 170,395 | 60 | 2,840 |  |  |
|  | Total | 175,419 | 61 |  |  |  |
| Distance 80 | Between Groups | 6,210 | 1 | 6,210 | 2,492 | ,120 |
|  | Within Groups | 149,484 | 60 | 2,491 |  |  |
|  | Total | 155,694 | 61 |  |  |  |
| Distance 90 | Between Groups | 6,189 | 1 | 6,189 | 2,467 | ,122 |
|  | Within Groups | 150,520 | 60 | 2,509 |  |  |
|  | Total | 156,710 | 61 |  |  |  |
| Distance 100 | Between Groups | 3,629 | 1 | 3,629 | 1,811 | ,183 |
|  | Within Groups | 120,242 | 60 | 2,004 |  |  |
|  | Total | 123,871 | 61 |  |  |  |
| Distance 110 | Between Groups | 1,662 | 1 | 1,662 | 1,274 | ,264 |
|  | Within Groups | 78,274 | 60 | 1,305 |  |  |
|  | Total | 79,935 | 61 |  |  |  |
|  | | **Sum of Squares** | **df** | **Mean Square** | **F** | **Sig.** |
| Distance 120 | Between Groups | 1,880 | 1 | 1,880 | 1,768 | ,189 |
|  | Within Groups | 63,797 | 60 | 1,063 |  |  |
|  | Total | 65,677 | 61 |  |  |  |
| Distance 130 | Between Groups | 2,320 | 1 | 2,320 | 2,528 | ,117 |
|  | Within Groups | 55,051 | 60 | ,918 |  |  |
|  | Total | 57,371 | 61 |  |  |  |
| Distance 140 | Between Groups | 5,229 | 1 | 5,229 | 4,473 | ,039 |
|  | Within Groups | 70,142 | 60 | 1,169 |  |  |
|  | Total | 75,371 | 61 |  |  |  |
| Distance 150 | Between Groups | 4,770 | 1 | 4,770 | 3,867 | ,054 |
|  | Within Groups | 74,004 | 60 | 1,233 |  |  |
|  | Total | 78,774 | 61 |  |  |  |
| Distance 160 | Between Groups | 2,973 | 1 | 2,973 | 2,714 | ,105 |
|  | Within Groups | 65,737 | 60 | 1,096 |  |  |
|  | Total | 68,710 | 61 |  |  |  |
| Distance 170 | Between Groups | 2,973 | 1 | 2,973 | 2,889 | ,094 |
|  | Within Groups | 61,737 | 60 | 1,029 |  |  |
|  | Total | 64,710 | 61 |  |  |  |
| Distance 180 | Between Groups | 2,683 | 1 | 2,683 | 3,172 | ,080 |
|  | Within Groups | 50,752 | 60 | ,846 |  |  |
|  | Total | 53,435 | 61 |  |  |  |
| Distance 190 | Between Groups | 1,517 | 1 | 1,517 | 1,528 | ,221 |
|  | Within Groups | 59,580 | 60 | ,993 |  |  |
|  | Total | 61,097 | 61 |  |  |  |
| Distance 200 | Between Groups | ,853 | 1 | ,853 | 1,407 | ,240 |
|  | Within Groups | 36,389 | 60 | ,606 |  |  |
|  | Total | 37,242 | 61 |  |  |  |
| Distance 210 | Between Groups | ,108 | 1 | ,108 | ,230 | ,633 |
|  | Within Groups | 28,086 | 60 | ,468 |  |  |
|  | Total | 28,194 | 61 |  |  |  |
| Distance 220 | Between Groups | ,263 | 1 | ,263 | ,749 | ,390 |
|  | Within Groups | 21,108 | 60 | ,352 |  |  |
|  | Total | 21,371 | 61 |  |  |  |
|  | | **Sum of Squares** | **df** | **Mean Square** | **F** | **Sig.** |
| Distance 230 | Between Groups | ,155 | 1 | ,155 | ,411 | ,524 |
|  | Within Groups | 22,683 | 60 | ,378 |  |  |
|  | Total | 22,839 | 61 |  |  |  |
| Distance 240 | Between Groups | ,076 | 1 | ,076 | ,249 | ,619 |
|  | Within Groups | 18,199 | 60 | ,303 |  |  |
|  | Total | 18,274 | 61 |  |  |  |
| Distance 250 | Between Groups | ,169 | 1 | ,169 | ,652 | ,423 |
|  | Within Groups | 15,509 | 60 | ,258 |  |  |
|  | Total | 15,677 | 61 |  |  |  |
