# Supplementary material for: Transcription factor Yin-Yang 2 alters neuronal outgrowth in vitro
Source: Cell Tissue Res. 2015 Sep 9;362(2):453–60. doi: 10.1007/s00441-015-2268-7 (PMC4657790; doi:10.1007/s00441-015-2268-7)
Supplement: Supplementary file 5 — Data from the multiple comparisons Bonferroni statistics of Fig. 2e. (DOC 248 kb) [file 441_2015_2268_MOESM4_ESM.doc]

**IBM SPSS Statistics Viewer**

**Multiple Comparisons**

**Bonferroni**

| **Dependent Variable** | **(I) Gruppe** | **(J) Gruppe** | **Std. Error** | **Sig.** |
| --- | --- | --- | --- | --- |
|  |  |  |  |  |
| Distance 20 | control | yy2 wt | ,544 | ,034 |
|  |  | yy2 mut | ,528 | 1,000 |
|  | yy2 wt | control | ,544 | ,034 |
|  |  | yy2 mut | ,528 | ,013 |
|  | yy2 mut | control | ,528 | 1,000 |
|  |  | yy2 wt | ,528 | ,013 |
| Distance 30 | control | yy2 wt | ,485 | ,037 |
|  |  | yy2 mut | ,472 | ,176 |
|  | yy2 wt | control | ,485 | ,037 |
|  |  | yy2 mut | ,472 | ,000 |
|  | yy2 mut | control | ,472 | ,176 |
|  |  | yy2 wt | ,472 | ,000 |
| Distance 40 | control | yy2 wt | ,503 | ,058 |
|  |  | yy2 mut | ,488 | ,084 |
|  | yy2 wt | control | ,503 | ,058 |
|  |  | yy2 mut | ,488 | ,000 |
|  | yy2 mut | control | ,488 | ,084 |
|  |  | yy2 wt | ,488 | ,000 |
| Distance 50 | control | yy2 wt | ,434 | ,001 |
|  |  | yy2 mut | ,422 | ,007 |
|  | yy2 wt | control | ,434 | ,001 |
|  |  | yy2 mut | ,422 | ,000 |
|  | yy2 mut | control | ,422 | ,007 |
|  |  | yy2 wt | ,422 | ,000 |
| Distance 60 | control | yy2 wt | ,466 | ,011 |
|  |  | yy2 mut | ,453 | ,036 |
|  | yy2 wt | control | ,466 | ,011 |
|  |  | yy2 mut | ,453 | ,000 |
|  | yy2 mut | control | ,453 | ,036 |
|  |  | yy2 wt | ,453 | ,000 |
| Distance 70 | control | yy2 wt | ,399 | ,046 |
|  |  | yy2 mut | ,387 | ,021 |
|  | yy2 wt | control | ,399 | ,046 |
|  |  | yy2 mut | ,387 | ,000 |
|  | yy2 mut | control | ,387 | ,021 |
|  |  | yy2 wt | ,387 | ,000 |
| Dependent Variable | (I) Gruppe | (J) Gruppe | Std. Error | Sig. |
|  |  |  |  |  |
| Distance 80 | control | yy2 wt | ,346 | ,052 |
|  |  | yy2 mut | ,337 | ,010 |
|  | yy2 wt | control | ,346 | ,052 |
|  |  | yy2 mut | ,337 | ,000 |
|  | yy2 mut | control | ,337 | ,010 |
|  |  | yy2 wt | ,337 | ,000 |
| Distance 90 | control | yy2 wt | ,281 | ,057 |
|  |  | yy2 mut | ,273 | ,003 |
|  | yy2 wt | control | ,281 | ,057 |
|  |  | yy2 mut | ,273 | ,000 |
|  | yy2 mut | control | ,273 | ,003 |
|  |  | yy2 wt | ,273 | ,000 |
| Distance 100 | control | yy2 wt | ,283 | ,323 |
|  |  | yy2 mut | ,275 | ,012 |
|  | yy2 wt | control | ,283 | ,323 |
|  |  | yy2 mut | ,275 | ,000 |
|  | yy2 mut | control | ,275 | ,012 |
|  |  | yy2 wt | ,275 | ,000 |
| Distance 110 | control | yy2 wt | ,320 | ,587 |
|  |  | yy2 mut | ,311 | ,019 |
|  | yy2 wt | control | ,320 | ,587 |
|  |  | yy2 mut | ,311 | ,000 |
|  | yy2 mut | control | ,311 | ,019 |
|  |  | yy2 wt | ,311 | ,000 |
| Distance 120 | control | yy2 wt | ,309 | ,479 |
|  |  | yy2 mut | ,301 | ,014 |
|  | yy2 wt | control | ,309 | ,479 |
|  |  | yy2 mut | ,301 | ,000 |
|  | yy2 mut | control | ,301 | ,014 |
|  |  | yy2 wt | ,301 | ,000 |
| Distance 130 | control | yy2 wt | ,283 | ,560 |
|  |  | yy2 mut | ,275 | ,004 |
|  | yy2 wt | control | ,283 | ,560 |
|  |  | yy2 mut | ,275 | ,000 |
|  | yy2 mut | control | ,275 | ,004 |
|  |  | yy2 wt | ,275 | ,000 |
|  |  |  |  |  |
| Dependent Variable | (I) Gruppe | (J) Gruppe | Std. Error | Sig. |
|  |  |  |  |  |
| Distance 140 | control | yy2 wt | ,271 | ,201 |
|  |  | yy2 mut | ,263 | ,007 |
|  | yy2 wt | control | ,271 | ,201 |
|  |  | yy2 mut | ,263 | ,000 |
|  | yy2 mut | control | ,263 | ,007 |
|  |  | yy2 wt | ,263 | ,000 |
| Distance 150 | control | yy2 wt | ,255 | 1,000 |
|  |  | yy2 mut | ,248 | ,001 |
|  | yy2 wt | control | ,255 | 1,000 |
|  |  | yy2 mut | ,248 | ,000 |
|  | yy2 mut | control | ,248 | ,001 |
|  |  | yy2 wt | ,248 | ,000 |
| Distance 160 | control | yy2 wt | ,220 | ,273 |
|  |  | yy2 mut | ,214 | ,002 |
|  | yy2 wt | control | ,220 | ,273 |
|  |  | yy2 mut | ,214 | ,000 |
|  | yy2 mut | control | ,214 | ,002 |
|  |  | yy2 wt | ,214 | ,000 |
| Distance 170 | control | yy2 wt | ,208 | ,068 |
|  |  | yy2 mut | ,202 | ,090 |
|  | yy2 wt | control | ,208 | ,068 |
|  |  | yy2 mut | ,202 | ,000 |
|  | yy2 mut | control | ,202 | ,090 |
|  |  | yy2 wt | ,202 | ,000 |
| Distance 180 | control | yy2 wt | ,185 | ,043 |
|  |  | yy2 mut | ,180 | ,038 |
|  | yy2 wt | control | ,185 | ,043 |
|  |  | yy2 mut | ,180 | ,000 |
|  | yy2 mut | control | ,180 | ,038 |
|  |  | yy2 wt | ,180 | ,000 |
| Distance 190 | control | yy2 wt | ,175 | ,030 |
|  |  | yy2 mut | ,170 | ,422 |
|  | yy2 wt | control | ,175 | ,030 |
|  |  | yy2 mut | ,170 | ,000 |
|  | yy2 mut | control | ,170 | ,422 |
|  |  | yy2 wt | ,170 | ,000 |
|  |  |  |  |  |
| Dependent Variable | (I) Gruppe | (J) Gruppe | Std. Error | Sig. |
|  |  |  |  |  |
| Distance 200 | control | yy2 wt | ,162 | ,016 |
|  |  | yy2 mut | ,158 | 1,000 |
|  | yy2 wt | control | ,162 | ,016 |
|  |  | yy2 mut | ,158 | ,001 |
|  | yy2 mut | control | ,158 | 1,000 |
|  |  | yy2 wt | ,158 | ,001 |
| Distance 210 | control | yy2 wt | ,154 | ,182 |
|  |  | yy2 mut | ,150 | ,529 |
|  | yy2 wt | control | ,154 | ,182 |
|  |  | yy2 mut | ,150 | ,004 |
|  | yy2 mut | control | ,150 | ,529 |
|  |  | yy2 wt | ,150 | ,004 |
| Distance 220 | control | yy2 wt | ,122 | ,035 |
|  |  | yy2 mut | ,119 | 1,000 |
|  | yy2 wt | control | ,122 | ,035 |
|  |  | yy2 mut | ,119 | ,004 |
|  | yy2 mut | control | ,119 | 1,000 |
|  |  | yy2 wt | ,119 | ,004 |
| Distance 230 | control | yy2 wt | ,109 | ,175 |
|  |  | yy2 mut | ,106 | ,556 |
|  | yy2 wt | control | ,109 | ,175 |
|  |  | yy2 mut | ,106 | ,004 |
|  | yy2 mut | control | ,106 | ,556 |
|  |  | yy2 wt | ,106 | ,004 |
| Distance 240 | control | yy2 wt | ,101 | ,447 |
|  |  | yy2 mut | ,098 | ,270 |
|  | yy2 wt | control | ,101 | ,447 |
|  |  | yy2 mut | ,098 | ,005 |
|  | yy2 mut | control | ,098 | ,270 |
|  |  | yy2 wt | ,098 | ,005 |
| Distance 250 | control | yy2 wt | ,107 | ,523 |
|  |  | yy2 mut | ,104 | ,155 |
|  | yy2 wt | control | ,107 | ,523 |
|  |  | yy2 mut | ,104 | ,003 |
|  | yy2 mut | control | ,104 | ,155 |
|  |  | yy2 wt | ,104 | ,003 |
